# Seasonality Modulates the Cellular Antioxidant Activity and Antiproliferative Effect of Sonoran Desert Propolis

**DOI:** 10.3390/antiox9121294

**Published:** 2020-12-17

**Authors:** Pablo Mendez-Pfeiffer, Efrain Alday, Ana Laura Carreño, Jorge Hernández-Tánori, Beatriz Montaño-Leyva, Jesús Ortega-García, Judith Valdez, Adriana Garibay-Escobar, Javier Hernandez, Dora Valencia, Carlos Velazquez

**Affiliations:** 1 Department of Chemistry-Biology, University of Sonora, Blvd. Luis Encinas y Rosales s/n, Hermosillo, Sonora C.P. 83000, Mexico; a209213202@unison.mx (P.M.-P.); efrain.alday@unison.mx (E.A.); anita_garza310385@hotmail.com (A.L.C.); martha.valdez@unison.mx (J.V.); adriana.garibay@unison.mx (A.G.-E.); 2 Department of Chemical Biological and Agropecuary Sciences, University of Sonora, Av. Universidad and Irigoyen, Caborca, Sonora C.P. 83600, Mexico; a211201591@alumnos.unison.mx (J.H.-T.); jesus.ortega@unison.mx (J.O.-G.); 3 Departamento de Investigacion y Posgrado en Alimentos, University of Sonora, Blvd. Luis Encinas y Rosales s/n, Hermosillo, Sonora C.P. 83000, Mexico; beatriz.montano@unison.mx; 4 Unidad de Servicios de Apoyo en Resolución Analítica, Universidad Veracruzana, Xalapa, Veracruz C.P. 91190, Mexico; javmartinez@uv.mx

**Keywords:** mixed poplar-type propolis, polyphenolic composition, antiproliferative effect, cellular antioxidant activity, seasonal effect

## Abstract

The main chemical composition and pharmacological potential of propolis from arid and semi-arid regions of the Sonoran Desert have been previously reported. Caborca propolis (CP), from an arid zone of the Sonoran Desert, has shown a polyphenolic profile that suggests a mixed plant origin, presenting poplar-type markers, as well as a 6-methoxylated flavonoid, xanthomicrol, characteristic of Asteraceae plants. In addition, CP has shown significant antioxidant properties and antiproliferative activity on cancer cells. In this study, we analyzed the influence of collection time on the chemical constitution, antiproliferative activity and protective capacity of CP against reactive oxygen species (ROS), by using HPLC–UV–diode array detection (DAD) analysis, 3-(4,5-dimethylthiazol-2-yl)-2,5-Dimethyltetrazoliumbromide (MTT) and 2,2-diphenyl-1-picryl-hydrazyl (DPPH) assays, as well as cellular antioxidant activity (CAA) assay on murine B-cell lymphoma M12.C3.F6 cells. HPLC–UV–DAD analyses of seasonally collected CP (one-year period) revealed quantitative differences among the most abundant CP constituents: pinocembrin, galangin, chrysin and pinobanksin-3-*O*-acetate. Though all seasonal samples of CP induced an antiproliferative effect in M12.C3.F6 cells, CP from autumn showed the highest inhibitory activity (IC_50_: 5.9 ± 0.6 µg/mL). The DPPH assay pointed out that CP collected in autumn presented the highest antioxidant potential (IC_50_: 58.8 ± 6.7 µg/mL), followed by winter (65.7 ± 12.2 µg/mL) and spring (67.0 ± 7.5 µg/mL); meanwhile, the summer sample showed a lesser antioxidant capacity (IC_50_: 98.7 ± 2.5 µg/mL). The CAA assay demonstrated that CP induced a significant protective effect against ROS production elicited by H_2_O_2_ in M12.C3.F6 cells. Pretreatment of M12.C3.F6 cells with CP from spring and autumn (25 and 50 µg/mL for 1 h) showed the highest reduction in intracellular ROS induced by H_2_O_2_ (1 and 5 mM). These results indicate that the antiproliferative effect and cellular antioxidant activity of CP are modulated by quantitative fluctuations in its polyphenolic profile due to its collection time.

## 1. Introduction

Cellular redox homeostasis relies on the orchestrated generation and neutralization of reactive oxygen species (ROS), which are unstable and ephemeral molecules produced during aerobic metabolism. ROS encompass oxidizing species, such as superoxide anion (O_2_^•−^), hydroxyl radicals (^•^OH), and non-radical molecules as hydrogen peroxide (H_2_O_2_). Under normal physiological conditions, slight and short-lived modulations of ROS are essential to regulate various signal pathways for different cellular processes, including development, proliferation and differentiation [1,2,3,4,5]. However, an unbalanced concentration of increasing intracellular levels of H_2_O_2_ could trigger the production of ^•^OH that would damage proteins, lipids and DNA, as well as activate signal transduction pathways that mediate pathophysiological processes [2,6,7]. Therefore, cellular machinery is adapted with molecular defense systems to counteract the generation of ROS by enzymatic, as well as non-enzymatic anti-oxidant processes [4,5]. 

Redox homeostasis is displaced to oxidative stress when the production of ROS has overpassed cellular antioxidant systems, resulting in an altered state associated to many inflammatory diseases [3,6]. A modified redox state has been described as a common feature in cancer cells, in which a higher production rate of mitochondrial ROS is proportional to an increased metabolic activity. This augmentation of ROS promotes the activation of signaling pathways and transcription factors essential for cancer survival, proliferation, and tumorigenesis [7]. Thus, in order to avoid the activation of death pathways and allow cancer progression, it is generally considered that cancer cells are more adapted with ROS scavenging systems than normal cells [1,7]. Though the inhibition of cellular ROS generation does not represent a feasible therapeutic target for the treatment of cancer, given the vital and dynamic role of ROS in normal cells, the ability to interfere and modulate the cellular redox state could disrupt some tumorigenic mechanisms and make cancer cells more susceptible to chemotherapeutic agents [8]. 

To prevent the negative effects of an unbalanced load of ROS, as well as to modulate signaling pathways upregulated in cancer cells, a supportive and protective cellular neutralization is further provided by dietary bioactive phytochemical molecules with antioxidant potential [3,9]. Polyphenolic compounds are secondary metabolites broadly distributed in edible plants and are known by their potential health benefits, including their ROS scavenging capacity [10,11]. Propolis is considered to be one of the richest sources of natural polyphenolic compounds, due to its abundance in flavonoids and phenolic acid derivatives, constituents that are associated to most biological properties of propolis. Propolis is produced by honeybees (*Apis mellifera*) from the environmentally available plant chemistry, resulting in a chemically diverse resinous substance, wherein compounds other than polyphenols provide bioactivity into the pharmacological potential of several propolis types. The broad array of biological activities of propolis include antioxidant, antibacterial, antiparasitic, antifungic, immunomodulatory activity and antiproliferative effects, among others [10,12,13,14,15,16,17]. At present, the seasonal influence on propolis production from dissimilar geographical areas, as well as derived from different plant origins, has been studied to establish a correlation between its pharmacological potential and the qualitative and quantitative fluctuations of propolis constituents throughout the year [18,19,20,21,22,23,24,25].

Propolis and polyphenols have demonstrated a great antioxidant activity, mostly by in vitro spectrophotometric assays, chemical methods based on the deactivation of free radicals, as well as either by hydrogen atom transfer (HAT) and single electron transfer (SET) mechanisms [26,27,28,29,30,31]. Nevertheless, the antioxidant effect assessed by chemical methods should be addressed in the biological context of oxidative stress, as well as by considering the availability, internalization and metabolism of the antioxidant compounds under physiological conditions. The cellular antioxidant activity (CAA) assay allows us to quantify the protective performance of antioxidant molecules in the cellular environment by measuring the ROS-oxidized fluorescent 2′,7′-dichlorofluorescin (DCF) impermeable probe [26,32,33,34]. CAA provides a more biological method that requires the cellular presence of an antioxidant compound, probe and the oxidant substance, but not in a simultaneous incubation, such as those carried out in chemical methods. Moreover, a fluorescent double staining of DCF with propidium iodide provides a strategy to analyze the viability, cell integrity and morphology of treated cells by flow cytometry [35]. 

In previous studies, we have reported the main chemical composition and pharmacological potential of propolis from the Sonoran Desert, collected in Caborca, Ures and Pueblo de Álamos regions. Caborca propolis (CP), an arid propolis sample from the Sonoran Desert, has been shown to be chemically constituted by polyphenolic compounds, including poplar chemical markers, such as pinocembrin, pinobanksin-3-O-acetate, chrysin and galangin [36], as well as 6-methoxylated lipophilic flavonoids, characteristic of the Asteraceae family [37], such as xanthomicrol [36,37,38]. In addition, CP has shown a significant antiproliferative effect on different cancer cell lines [36], as well as free radical scavenging activity, by the use of a chemical colorimetric assay (DPPH) [39]. In order to investigate the seasonal influence on the chemical composition and pharmacological potential of methanolic extracts of CP from the Sonoran Desert, we qualitatively and quantitatively analyzed the polyphenolic profile of seasonal samples collected from winter to fall (2014-2015) by HPLC-UV-DAD, followed by in vitro determination of the antiproliferative effects, ROS scavenging potential (DPPH assay), and cellular anti-oxidant activity (CAA) of CP collected throughout the year. In this study, we found that the antiproliferative activity and cellular protective effect of CP against ROS are greatly influenced by seasonal effects on its polyphenolic composition, providing new insights into propolis research.

## 2. Materials and Methods

### 2.1. Chemical Reagents

Dimethyl sulfoxide (DMSO), 3-(4,5-dimethylthiazol-2-yl)-2,5-Dimethyltetrazoliumbromide (MTT), 2,2-diphenyl-1-picryl-hydrazyl (DPPH), 2′,7′-dichlorofluorescin diacetate (DCFH-DA; ≥ 97.0%), Dulbecco’s Modified Eagle’s Medium (DMEM), NaHCO_3_ (≥ 99.5%), L-asparagine (98%), L-arginine monohydrochloride (≥ 98%), L-glutamine solution (200 mM), sodium pyruvate solution (100 mM), penicillin-streptomycin solution (1000 U/1 U per mL), ethanol, methanol (analytical grade), caffeic acid, phenethyl alcohol, *p*-toluene sulphonic acid, propidium iodide (PI), hexane, ethyl acetate, as well as HPLC grade methanol and formic acid (FA), were purchased from Sigma-Aldrich Co. (St. Louis, MO, USA). Hydrogen peroxide (30%) was purchased from J. T. Baker Chemicals. Fetal bovine serum (FBS) was purchased from Gibco (Carlsbad, CA, USA). HPLC-grade water (18 mΩ) was prepared by a Milli-Q A10 purification system (Millipore Corp.). Pinocembrin (≥ 95%), chrysin (≥ 95%), galangin (≥ 95%) and pinobanksin-3-*O*-acetate (≥ 95%) were purified from propolis collected in Ures, Sonora, Mexico (N 29° 27.181′, W 110° 23.398) by chromatographic isolation procedures over silica gel 60 (0.015–0.040 mm; Merck KGaA), using progressive proportions of ethyl acetate in hexane as the mobile phase (Tedia Company). Purity was assessed by HPLC–UV–DAD, as well as by ^1^H NMR spectra analysis, using deuterated chloroform or acetone (Sigma-Aldrich Co, St. Louis, MO, USA) in an Agilent 400/54 NMR spectrometer, operating at 400.02 MHz or ^1^H and 100.59 MHz for ^13^C (Agilent VnmrJ software package 3.2, Agilent Technologies, Inc. 2011, Santa Clara, CA, USA) [19]. Caffeic acid phenethyl ester (CAPE) was synthesized according to the esterification of caffeic acid and phenethyl alcohol, based on the procedure described by Grunberger et al. [35,40]. 

### 2.2. Propolis Collection and Methanolic Extraction

Sonoran Desert propolis was collected from twelve hives of Tecolote farm (N 31°02.18′, W 112°02.58′), located in the arid region “El Arenoso”, between Caborca and Altar municipalities, Sonora, Mexico. Caborca propolis (CP) was collected over the four seasons of the year, from winter 2014 to fall 2015. Previous studies have shown the chemical stability of Sonoran propolis collected throughout the years [19,20,36]. Other studies have reported no qualitative changes in the chemical composition of propolis after several years of storage at freezing temperatures [41]. At the beginning of each season, standard colony hives were prepared with propolis wooden traps (59 × 37 cm) lined with polyethylene mesh with a pore size of 1.0 mm^2^ and then covered with colony lid. Propolis traps were removed at the end of each season and were frozen (−80 °C) to allow propolis harvesting. The seasonal samples of CP (10 g) were cut into small pieces and extracted with methanol (50 mL) for three days, then filtered to concentrate the methanolic extracts under reduced pressure. Once the methanol was evaporated from the propolis methanolic extract, we removed the remaining waxes in CP by washing the obtained dry extracts with hexane (1:3 p/v), as previously published by our research group [19,20,36]. Seasonal samples of CP were stored in the dark at −20 °C until analysis.

### 2.3. Polyphenolic Profile Analyses by HPLC–UV–DAD

Seasonal samples of CP were analyzed on an Agilent 1290 Infinite series. The column (Zorbax Eclipse Plus C18 4.6 × 100 mm 2.5 Micron) was eluted using a binary gradient formed by 5% FA in H_2_O (solvent A) and methanol (solvent B) at a flow rate of 1 mL/min. The gradient program adopted was as follows: time 0–2 min, A 70%, B 30%; 5 min, A 60%, B 40%; 10 min, A 55%, B 45%; 50 min, A 40%, B 60%; 80 min, A 20%, B 80%, followed by washing and re-equilibrating of the column. Samples were prepared at 1 mg/mL in MeOH and filtered through nylon membrane (0.22 µm). The UV spectra were recorded at a 200–600 nm range using a diode array detector (DAD). Detection of compounds was monitored and recorded at 280 nm [20,36]. The chemical standards of poplar compounds (pinocembrin, galangin, chrysin and pinobanksin-3-O-acetate) were prepared at 400 µg/mL to quantify poplar compounds in seasonal samples of CP; the calibration curves of each chemical standard were prepared from 0 to 80 µg/mL. Chromatograms of seasonal samples of CP were recorded at 280 nm.

### 2.4. Free Radical Scavenging Activity (DPPH)

Free radical scavenging activity was measured by DPPH assay as previously reported by Usia et al. [42] with some modifications. Seasonal samples of CP were dissolved in methanol (100 µL) and were mixed with 300 µM DPPH solution (100 µL) into a flat 96-well plate (Costar, Corning, Corning, NY, USA) and incubated in the dark for 30 min. The absorbance was measured at 517 nm by an ELISA plate reader (Multiskan GO, Thermo Scientific, Waltham, MA, USA). The free radical scavenging activity of seasonal samples of CP was measured at different concentrations (100 µg/mL, 50 µg/mL and 25 µg/mL). Ascorbic acid was used and prepared (70 µM) as an antioxidant standard, according to previous studies [20]. Free radical scavenging activity is reported as a percentage decrease with respect to ascorbic acid control.

### 2.5. Cell Culture and Antiproliferative Activity Assay

The M12.C3.F6 cell line (murine B-cell lymphoma) was provided by Emil R. Unanue, Department of Pathology and Immunology, Washington University in St. Louis, MO, USA. The B-cell lymphoma M12.C3.F6 cell line was used as a cellular model due to its culture characteristics, such as non-adherent features, as well as its sensitivity to cytotoxic drugs and susceptibility to oxidative stress induction with H_2_O_2_ [35,36,43]. M12.C3.F6 cells were cultured in DMEM supplemented with 5% heat inactivated FBS. With the aim to determine the susceptibility of M12.C3.F6 cells to CP, and the inhibitory effect of treatments on cell proliferation, we carried out a standard MTT assay [44] with some modifications [36]. Initially, M12.C3.F6 cells were seeded (1 × 10^4^ cells, 50 μL) into each cell of a flat-bottom 96-well plate (Costar, Corning). After 24 h of incubation at standard culture conditions (37 °C under an atmosphere of 5% CO_2_), equal aliquots (50 μL) of DMEM containing different concentrations of each CP seasonal sample were added. Cells were incubated for 48 h. During the last 4 h of treatment, 10 μL of an MTT solution (5 mg/mL) were added to each well. Acidic isopropyl alcohol (100 µL; 0.3%) was used to dissolve the formazan crystals. The absorbance was measured by an ELISA plate reader (Multiskan EX, ThermoLabSystem, Waltham, MA, USA), using a test wavelength of 570 nm with a reference wavelength of 650 nm. The antiproliferative activity of seasonal samples of CP is reported as the IC_50_ value, obtained by linear regression analyses of each seasonal CP treatment.

### 2.6. Cellular Anti-Oxidant Activity Assay (CAA)

The cellular redox state was measured in M12.C3.F6 cells treated with seasonal samples of CP, according to the intracellular ROS level detected by using the cellular antioxidant activity assay (CAA) adapted to flow cytometry [32,33] with some modifications [35]. A cellular suspension of M12.C3.F6 cells (200,000 cells/mL) was placed in each well of a 6-well plate (Costar, USA). After 24 h incubation at standard culture conditions (37 °C in a 5% CO_2_ atmosphere), an aliquot (1 mL) containing two different concentrations (25 and 50 µg/mL) of seasonal samples of CP was added. CAPE (5 µM) was used as a control, according to its protective effect against ROS, as previously demonstrated in M12.C3.F6 cells [35]. 

After 1-h treatment, cells were harvested and washed twice (575× *g*, 7 min, 4 °C) with cold PBS (pH 7.2). Then, cells were incubated with DCFH-DA (1 µM in PBS; pH 7.2) protected from light for 30 min at 37 °C in a 5% CO_2_. The DCFH-DA probe precursor is intracellularly de-esterified to DCFH_2_, a molecule that is oxidized to the fluorescent probe DCF by cellular ROS [32,33,34]. Then, cells were washed twice with cold PBS, and cellular oxidative stress was induced with two different concentrations of H_2_O_2_ (1 and 5 mM) for 10 min at room temperature. H_2_O_2_ at the highest concentration tested (5 mM) did not affect cell viability. After 10 min with H_2_O_2_, the reaction was diluted out with cold PBS, and cells were washed once with cold PBS, and incubated with Propidium Iodide (PI) (1 µg/mL) for 10 min at room temperature in the dark. 

Finally, cells were washed and resuspended in cold PBS and immediately analyzed by flow cytometry (FACS Canto II, Becton Dickinson, CA, USA). Only metabolically active cells with uncompromised cell membranes were analyzed for each condition, according to DCF and PI fluorescence. ROS production in M12.C3.F6 was estimated by a displacement in the mean fluorescence intensity (MFI) of DCF in comparison to DMSO under the same oxidative conditions (0, 1 and 5 mM H_2_O_2_) [35]. CAA expressed, as a percentage, was calculated according to the MFI values of DCF (ROS production) in M12.C3.F6 cells treated with the DMSO dissolvent control.

CAA % = 100 − [MFI _treatment_][100]/[MFI_DMSO_]

### 2.7. Statistical Analyses

The results shown were obtained by at least three independent experiments carried out in triplicate. CAA analysis was performed at least by two independent experiments at each tested condition. Data were graphed (mean ± standard deviation) using Prism 5 software v5.01 (2007). Differences in medians (*p <* 0.05) between the CAA of each CP treatment condition group to the DMSO control were analyzed by means of a non-parametric Mann–Whitney *U* test. In addition, a Kruskal–Wallis test was used to compare differences in population distribution amongst CP treatments (*p <* 0.05), according to each concentration (25 and 50 µg/mL). Antiproliferative data were analyzed by one-way ANOVA with Tukey’s comparison post-test. DPPH data were analyzed by two-way ANOVA with a Bonferroni post-test. Differences with a value of *p* less than 0.05 were considered significant (Prism 5 software v5.01, 2007). 

## 3. Results

**Figure 1:**
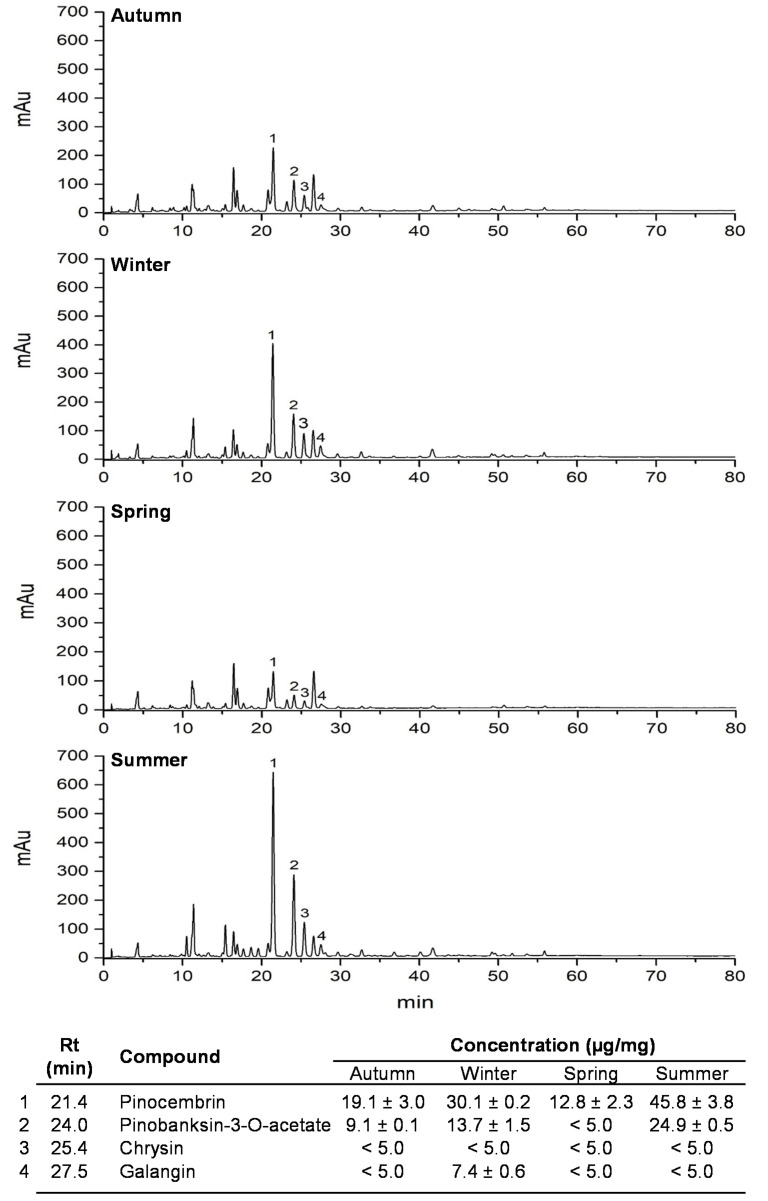
Polyphenolic profile of seasonal samples of Caborca propolis (CP) recorded at 280 nm. All seasonal samples were prepared at 1 mg/mL in MeOH. CP poplar-type compounds are enlisted within the figure. Pinocembrin (1), pinobanksin-3-*O*-acetate (2), chrysin (3) and galangin (4) were identified and assigned in the chromatogram of each seasonal sample, according to retention time (R*_t_*) and UV_max_ absorption spectra of analyzed chemical standards. A calibration curve of each of the chemical standards was prepared from 80 to 0 µg/mL. All values represent the mean of a least three independent determinations ± standard deviation. Chromatograms of seasonal samples of CP and compounds were recorded at 280 nm.

We qualitatively and quantitatively analyzed the polyphenolic profile of CP through the seasonal samples collected over a 1-year period. According to the designed HPLC–UV–DAD method, CP showed a similar qualitative constitution among the seasonal samples, mainly composed by phenolic acids and flavonoids (Figure 1). By using the previously identified poplar-type markers in CP (pinocembrin, chrysin, galangin and pinobanksin-3-*O*-acetate) [36], we observed quantitative and significant differences in those compounds among CP seasonal samples. Pinocembrin, together with pinobanksin-3-O-acetate, were the poplar markers that showed the most evident seasonal effects in CP (Figure 1). Pinocembrin, as well as pinobanksin-3-*O*-acetate, presented their highest concentration in summer samples (45.8 ± 3.8 and 24.9 ± 0.5 µg/mg, respectively), followed by winter (30.1 ± 0.2 and 13.7 ± 1.5 µg/mg, respectively); meanwhile, their presence was significantly lower in autumn (19.1 ± 3.0 and 9.1 ± 0.1 µg/mg, respectively), and even lower in the spring seasonal samples (12.8 ± 2.3 and < 5.0 µg/mg, respectively). These results indicated that the concentration of poplar-type flavonoids is quantitatively affected in CP by seasonal shifts, even though there is a qualitative resemblance found in the chemical profile of CP among seasonal samples (Figure 1). 

Once we found quantitative variations in the polyphenolic profile of seasonal samples of CP, we determined whether those changes in the relative abundance of CP constituents would inflict modifications on the pharmacological potential of CP. By using MTT assays, we observed that the samples collected over the four seasons of the year induced a significant antiproliferative effect in M12.C3.F6 cells (Table 1) after 48 h treatment (*p* < 0.05), in comparison to the dissolvent control (DMSO). CP produced during the autumn season induced the highest significant inhibitory effect (*p* < 0.05) in M12.C3.F6 cell proliferation (IC_50_: 5.9 ± 0.6 µg/mL), in comparison to spring (9.2 ± 1.3 µg/mL), summer (9.5 ± 0.4 µg/mL) and winter (12.1 ± 0.5 µg/mL) propolis (Table 1). These data suggest that quantitative variations in CP chemical constituents have a direct effect on the antiproliferative activity of CP. Caffeic acid phenethyl ester (CAPE), a constituent of poplar-type propolis, was used as a positive antiproliferative drug in M12.C3.F6 cells (IC_50_: 1.4 ± 0.1 µM), according to previous studies [35,36,40]. 

**Figure 2:**
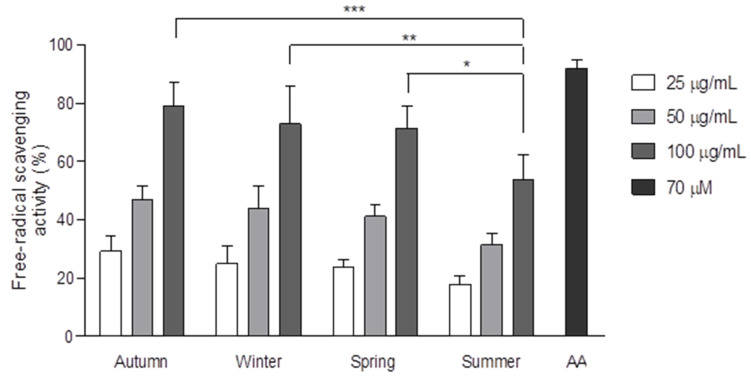
Free radical scavenging activity of seasonal samples of CP determined by 2,2-diphenyl-1-picryl-hydrazyl (DPPH) assay. Different concentrations of each seasonal methanolic extract of CP (25, 50 and 100 µg/mL) were assessed by DPPH. Ascorbic acid (AA) (70 µM) was used as antioxidant standard according to previous studies [20,39]. The results shown are the mean of at least three independent experiments performed by triplicate ± standard deviation. Data were analyzed by two-way ANOVA followed by a Bonferroni test. Significant differences among the seasonal samples are indicated. *** *p <* 0.001 of significance from the corresponding antioxidant effect of a CP seasonal sample to summer CP treatment (100 µg/mL). ** *p <* 0.01 of significance from the corresponding antioxidant effect of a CP seasonal sample to summer CP treatment 100 µg/mL). * *p <* 0.05 of significance from the corresponding antioxidant effect of a CP seasonal sample to summer CP treatment (100 µg/mL).

The influence of seasonal effects on free radical scavenging capacity was assessed by DPPH assay (Figure 2). CP collected in autumn, winter and spring presented similar antioxidant potentials (IC_50_: 58.8 ± 6.7, 65.7 ± 12.2 and 67.0 ± 7.5 µg/mL, respectively). CP collected in summer showed a lesser antioxidant capacity among the seasonal samples (IC_50_: 98.7 ± 2.5 µg/mL). Statistical differences were found among the antioxidant activity of CP seasonal samples at 100 µg/mL; CP from autumn, winter and spring were able to reduce DPPH radical up to 80% at 100 µg/mL, while CP from summer reduced 54% of the DPPH radical at the same concentration (*p <* 0.05). Ascorbic acid (AA) was used as antioxidant control (91.6% at 70 µM, Figure 2) in the DPPH assay according to previous studies [20,39]. At 100 µg/mL, no significant differences were found in the antioxidant capacity between CP from autumn, winter and spring and ascorbic acid (70 µM), indicating a high free radical scavenging capacity in those seasonal samples of CP.

**Figure 3:**
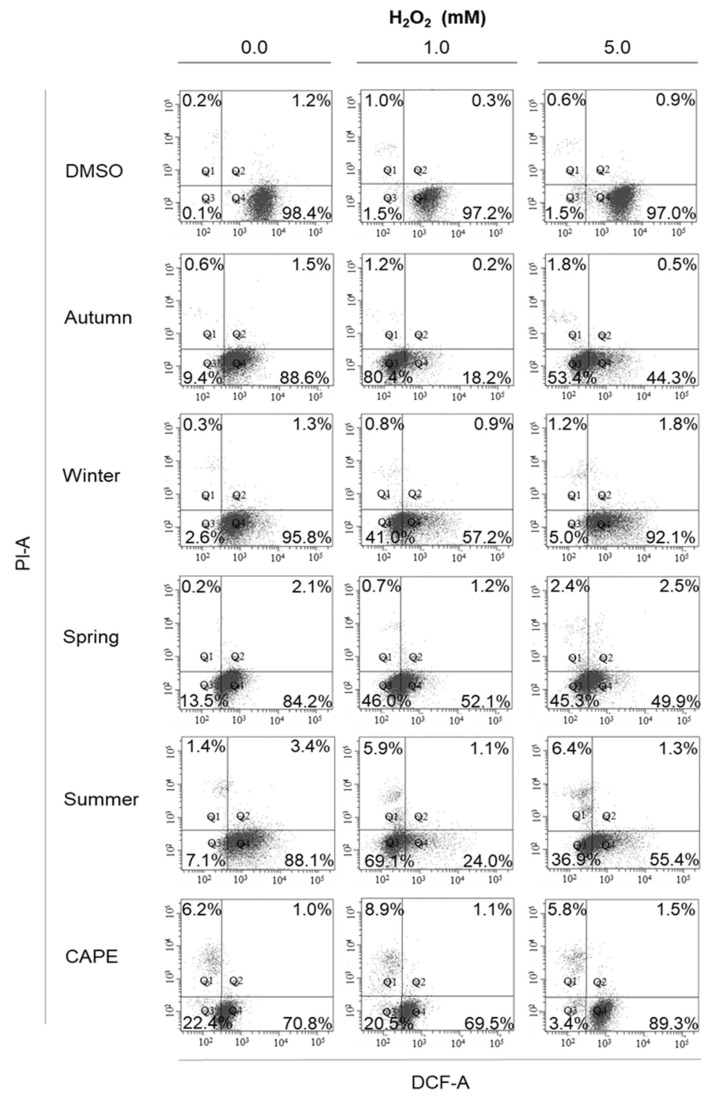
Morphological analysis of M12.C3.F6 cells treated with seasonal samples of CP (25 µg/mL) and H_2_O_2_ (0, 1 and 5 mM). Cellular antioxidant activity (CAA) was determined on morphologically and metabolically viable cells by forward scatter (FSC) and side scatter (SSC) parameters, according to treated cells, as well as by exclusion of PI staining. Quadrants Q3 and Q4 encompass viable and metabolically active cells under each tested condition (wherein at least 90–95% of cells were viable). Caffeic acid phenethyl ester (CAPE) was included as a positive control for CAA assays. Dot plots depicting morphological analysis are representative of at least three independent experiments.

In order to evaluate the antioxidant potential of seasonal samples of CP against biological ROS in the cellular environment, a cellular antioxidant activity (CAA) assay was performed in M12.C3.F6 cells under different oxidative stress conditions (0, 1 and 5 mM H_2_O_2_). Given the methodological characteristics of the assay, we analyzed only the metabolically active cell population with membrane integrity. By considering DCF and PI fluorescence, none of the CP seasonal treatments, together with H_2_O_2_ treatment conditions, significantly affected cell viability or membrane integrity during the 1 h period (Figure 3, Q1 and Q2). Dot plots depicted in Figure 3 illustrate the DFC and PI fluorescence distribution among M12.C3.F6 cells treated with CP and H_2_O_2_ (0, 1 and 5 mM), wherein lower quadrants (Q3 and Q4) comprise viable cells (negative to PI). At least 90 to 95% of M12.C3.F6 cells (Q3 and Q4) were viable and morphologically similar to those cells pretreated with dissolvent control (DMSO). Cellular morphology distribution of treated M12.C3.F6 cells with CP seasonal samples and H_2_O_2_ (1 and 5 mM) did not appear to be affected, in comparison to the forward scatter (FSC) and side scatter (SSC) parameters of cells treated with DMSO and H_2_O_2_ (data not shown). CAPE was included as a CAA control based on previous studies [35]. Interestingly, a more homogeneous distribution of the cell population was observed in M12.C3.F6 cells treated with seasonal samples of CP and H_2_O_2_, similar to CAPE, showing a remarkable fluorescence displacement from Q4 to Q3 (Figure 3) in comparison to the dissolvent control (DMSO) and H_2_O_2_. These results suggest that CP not only induced CAA on M12.C3.F6 cells by reducing the MFI values (DCF) of intracellular ROS after H_2_O_2_-stress induction, but also morphological protection against oxidative stress.

**Figure 4:**
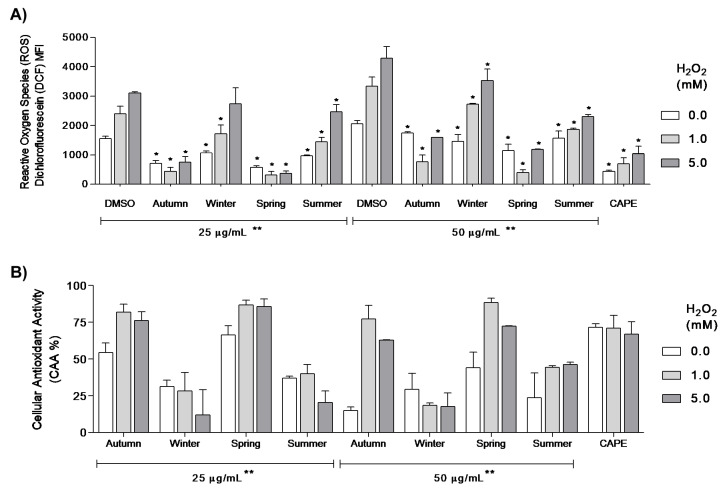
Cellular antioxidant activity (CAA) of seasonal samples of CP (25 and 50 µg/mL) in M12.C3.F6. Oxidative stress was induced with H_2_O_2_ (1 and 5 mM). M12.C3.F6 cells were treated for 1 h with seasonal samples of CP, then harvested, washed and incubated with DCFH-DA (1 µM), then with H_2_O_2_ (1 and 5 mM), and stained with PI (1 µg/mL). (**A**) The effect of seasonal samples of CP (25 and 50 µg/mL) on reactive oxygen species (ROS) production in M12.C3.F6 cells treated with 0, 1 and 5 mM H_2_O_2_, according to Dichlorofluorescein (DCF) mean fluorescence intensity (MFI) in fluorescence intensity arbitrary units (FIAU). (**B**) CAA of seasonal samples of CP in M12.C3.F6 cells is expressed in percentage, as obtained by comparison to ROS production (DCF MFI) in M12.C3.F6 treated with dimethyl sulfoxide (DMSO) dissolvent control under oxidative stress with 1 and 5 mM H_2_O_2_. The displayed results are the mean of at least two independent experiments ± standard deviation. CAPE (5 µM) was included as positive control for CAA assays. Data were analyzed by a Mann–Whitney *U* non-parametric test to compare each CP treatment condition or CAPE group to DMSO control (25 or 50 µg/mL). Significant differences between CAA of the seasonal samples to DMSO control are indicated * (*p <* 0.05). A Kruskal–Wallis test was used to compare differences in population distribution amongst all treatments on each tested concentration (25 and 50 µg/mL), significant differences (*p <* 0.05) were found in CAA amongst groups (25 and 50 µg/mL) and are marked as **.

According to the mean fluorescence intensity (MFI) of DCF, as a measure of ROS intracellular burden in M12.C3.F6 cells, pretreatment with methanolic extracts of seasonal samples of CP (25 µg/mL, for 1 h) induced a significant reduction in ROS burden at basal conditions, in comparison to the DMSO control, according to a Mann–Whitney *U* test (*p <* 0.05). Particularly, CP from autumn and spring (25 µg/mL, for 1 h) induced a 2.2- to 2.7-fold reduction in ROS at basal conditions (CAA: 54.4 ± 6.4% and 66.3 ± 6.4%, respectively), in comparison with DMSO treatment (*p <* 0.05, Figure 4) and basal M12.C3.F6 control (DCF MFI: 2203 ± 230 FIAU), suggesting its high antioxidant potential under normal cellular redox conditions, prior to the oxidative stress induction with H_2_O_2_ (Figure 4). Meanwhile, the control compound CAPE (5 µM) induced a 3.5-fold reduction in ROS at basal conditions (71.5 ± 1.5% in CAA). No significant differences were found between CP from spring and CAPE at basal conditions, according to a Kruskal–Wallis test followed by Dunn’s multiple comparison test (Figure 4). CP collected in autumn and spring (25 µg/mL) showed the most evident protective effect against oxidative stress, being able to significantly decrease the MFI values of ROS observed in cells treated with DMSO under 1 mM H_2_O_2_ (5.5 and 7.7 times, respectively) and 5 mM H_2_O_2_ (4.2 and 8.6 times, respectively; Figure 4A). CP collected in spring and autumn presented the highest CAA (from 76.0 to 90.5%) under oxidative stress induction with H_2_O_2_ (1 and 5 mM; Figure 4B). No significant differences were found between the CAA of CP from autumn and spring to CAPE (up to 79.6% of CAA) at tested conditions (1 and 5 mM H_2_O_2_; Figure 4A,B). A Kruskal–Wallis test revealed significant differences among the CAA percentage induced by seasonal samples at 25 µg/mL treatment. These results indicate that the CAA of CP is seasonally influenced (Figure 4).

At 50 µg/mL, all methanolic extracts of seasonally collected CP induced a significant reduction in basal ROS levels (0.0 mM H_2_O_2_) in M12.C3.F6 cells (Figure 4). Once the oxidative stress was induced with 1 and 5 mM H_2_O_2_, the spring sample showed the highest protective effect by reducing ROS levels by 8.6 and 3.6 times, respectively, followed by autumn CP, which, in turn, decreased ROS levels by 4.4 and 2.7 times, similar to CAPE (3.5- and 3.0-fold reduction, under 1 and 5 mM H_2_O_2_ treatment, respectively), indicating a high protective effect at this concentration (50 µg/mL) under the same conditions, in comparison with DMSO treatment (Figure 4A). At 1 mM H_2_O_2_, the CAA of CP collected during spring and autumn (50 µg/mL) was as high as 88.3% and 77.2%, respectively; meanwhile, at 5 mM H_2_O_2_ their CAA was 72.3% and 63.0%, respectively, an antioxidant effect as high as that induced by CAPE (5 µM; Figure 4B). In general, Mann–Whitney *U* test revealed that pretreatment with seasonal samples of CP (50 µg/mL, for 1 h) induced a significant CAA (*p <* 0.05). CP from spring and autumn induced the highest protective effect in M12.C3.F6 cells against different conditions of oxidative stress (1 and 5 mM H_2_O_2_), at both 25 and 50 µg/mL treatments. CP collected in summer and winter seasons induced a significant CAA; however, these CP seasonal samples were significantly less active than those from spring and autumn, according to the Kruskal–Wallis test (*p <* 0.05; Figure 4). These results suggest that the protective effect of CP against H_2_O_2_ oxidative stress induction is directly related to the ROS scavenging potential of cellular internalized CP constituents, and this is influenced by both collection season and propolis concentration.

## 4. Discussion

In a previous study, propolis from the arid region of Caborca, Sonora (CP) in the Sonoran Desert, particularly collected from autumn to spring, showed significant antiproliferative activity in human and murine cancer cell lines, as well as a significant free radical scavenging activity, measured by a chemical assay [36,39]. In this study, in order to further characterize propolis from this arid region, we used the cellular antioxidant activity (CAA) assay to analyze whether the antioxidant potential of CP would induce a cellular protective effect against ROS under oxidative stress conditions. In addition, with the aim of determining the influence of seasonal shift on the chemical composition and pharmacological potential of CP, we analyzed the polyphenolic profile, antioxidant activity by DPPH and CAA, and the antiproliferative effect of seasonal samples collected over a 1-year period.

The polyphenolic profile of CP evidenced quantitative fluctuations, mostly between poplar-type constituents among the seasonal samples. CP collected during the summer season showed the highest concentration of pinocembrin and pinobanksin-3-*O*-acetate amongst the seasonal samples. In previous studies, propolis from the semi-arid region of Ures (UP), a propolis type mainly derived from poplar tree exudates (*P. fremontii*), presented greater amounts of pinocembrin (218.4 ± 1.5 mg/g) than the sample collected from Caborca (60.6 ± 1.2 mg/g). In addition, the sample from UP (at 100 µg/mL) showed a lower antioxidant capacity (23.4 ± 1.5%) than CP at the same concentration (86.0 ± 0.3%). Furthermore, pinocembrin was reported as a flavonoid with no significant free radical scavenging potential (IC_50_: > 100 µM) [36,39]. This information suggests that antioxidant compounds of CP from summer could be diluted out with greater amounts of pinocembrin, thereby explaining the lesser free radical scavenging activity of the summer sample in comparison with the other seasonal samples (Figure 2).

The pharmacological potential of polyphenolic compounds is broadly described, including their antiproliferative effect and ROS scavenging capacity [10,11]. In addition, an association between intracellular ROS levels and cancer has been demonstrated, wherein increasing levels of ROS could promote the activation of signaling pathways and transcription factors implicated in cancer cell proliferation, as well as tissue invasion, metastasis and angiogenesis [1,7,45,46]. Hence, the antiproliferative effect of compounds that modulate intracellular ROS levels should be studied with the aim to characterize their bioactivity.

CP has been previously reported as an antiproliferative polyphenolic matrix from the Sonoran Desert [36]. Herein, a seasonal effect was observed in the antiproliferative effect of CP in B-cell lymphoma cell line M12.C3.F6, wherein the autumn sample was the most active against M12.C3.F6 cell proliferation. Interestingly, the mean antiproliferative activity index (IC_50_) from autumn, in comparison to a previously studied sample from November to March, showed corresponding bioactivity (IC_50_: 4.1 µg/mL), suggesting that the inhibitory effect of CP on cancer cell proliferation is mostly due to the phenology of persistently selected plants by honeybees [36]. In previous studies of propolis from the Sonoran Desert, we found that the antiproliferative effect of UP in human and murine cancer cell lines, such as M12.C3.F6, was clearly influenced by collection time [19,20]. These seasonal variations are additionally supported by recent studies that have established a correlation between total phenolic compounds in Brazilian red propolis and meteorological parameters throughout the year, such as rainfall intensity, relative humidity and solar radiation, conditions that modulate the biosynthesis of polyphenols in selected plants, and therefore determine the pharmacological potential of seasonal propolis [18]. Other studies also reported seasonal differences in the antimicrobial activity of red propolis collected during seasons with rainy and dry periods, observing quantitative differences in chemical composition and supporting the variability of propolis biological activities throughout the year [47]. The CAA assay evidenced a seasonal influence on ROS scavenging potential by CP under oxidative stress conditions. CP from spring and autumn induced the highest protective effect against oxidative stress in M12.C3.F6 cells, by decreasing the induced ROS levels, as well as by preserving a homogeneous morphology distribution of cell population, in comparison with DMSO, and CP samples from summer and winter. Noteworthily, CAA of CP from spring and autumn (25 and 50 µg/mL) was similar to that obtained with the antioxidant-positive control, CAPE (5 µM), at both 1 and 5 mM H_2_O_2_ conditions, indicating the high antioxidant potential of CP. The CAA results are in agreement with those variations observed in the polyphenolic profile of seasonal samples, wherein CP from autumn and spring has the lowest amount of poplar flavonoids amongst the seasonal samples, as well as the highest CAA, indicating that CP has a differential antioxidant potential clearly influenced by seasonal effect.

The antioxidant activity of aqueous poplar bud (*Populus nigra*) extract, as well as that of its main phenolic compounds, has been determined on human fibroblasts by CAA assay. *P. nigra* exudates showed moderate antioxidant properties, reducing DCF fluorescence by up to 53% at a 200 ug/mL treatment, whereas pinocembrin and pinobanksin reduced DCF fluorescence by 39 and 27% at 200 uM, respectively [48]. This moderate antioxidant effect of *P. nigra* extract is in agreement with our results, since *P. nigra* exudates are described as the main botanical source of European poplar-type propolis [13,48], indicating that poplar compounds obtained from *P. fremontii* buds are not necessarily correlated to those antioxidant compounds present in CP. In this study, we found that the DPPH assay was not able to discern between the antioxidant potential of CP collected during spring, autumn and winter (Figure 2). Similar discrepancies between chemical and biological antioxidant assays had been previously reported in other studies [48]. According to the IC_50_ values obtained by the DPPH assay, CP from winter is similar or slightly more active than that from spring (65.7 ± 12.2 and 67.0 ± 7.5 µg/mL, respectively), although this difference was not significant. Via the DPPH assay, we determined that the free radical scavenging potential of CP for autumn, winter and spring (at 50 µg/mL) was 46.8 ± 4.7, 43.7 ± 7.8 and 41.1 ± 4.2 %, respectively. By using the CAA assay, we found that CP from winter exerted a significantly lower protective effect against biological ROS in M12.C3.F6 cells, in comparison to the antioxidant potential induced by CP from autumn and spring. At 50 µg/mL and at 1.0 mM H_2_O_2_, the CAA of CP from autumn, winter and spring resulted in 81.9 ± 5.5, 28.4 ± 12.4 and 86.7 ± 3.5 %, respectively; meanwhile, CAA was 76.0 ± 6.2, 12.0 ± 17.0 and 85.8 ± 5.0 %, respectively, at the same concentration and 5.0 mM H_2_O_2_. Herein, CAA provided a sensitive strategy that allowed us to deeply analyze the seasonal effect on ROS scavenging potential of CP into biological context.

Ascorbic acid is one of the most used antioxidant control compounds in the DPPH assay; however, previous studies have demonstrated that its efficiency in a chemical assay could not be properly related to that obtained by CAA [35]. CAA has been developed with the advantages of both chemical and biological methods to determine, under oxidative stress induction, the protective potential of a certain substance to neutralize ROS production inside the cell. An important feature of CAA is that cells are not simultaneously exposed to an antioxidant treatment, a probe and an oxidative stress inducer (H_2_O_2_), which affords a more stringent method. Therefore, in order to pharmacologically characterize a bioactive substance, it is important to analyze its antioxidant potential in assays that consider the cellular environment, such as CAA.

Recently, we have determined the plant origin of propolis collected from the semi-arid region of Ures (UP), wherein *P. fremontii* exudates are the main botanical source. However, the biological properties of those propolis samples, specifically their antiproliferative effect on cancer cells, suggested the slight contribution of other bioactive resinous materials from different sources other than poplar trees, which enhanced their pharmacological properties [19]. Here, CP, a Sonoran Desert propolis sample from the arid region of Caborca, exhibited poplar compounds probably obtained from *P. fremontii*, as well as the previously identified xanthomicrol, a lipophilic flavonoid not biosynthesized by poplars, suggesting its mixed plant origin [36]. Therefore, the seasonal effect on CP constitution could also be related to the contribution of other plants. Moreover, the bioactivity of CP collected in spring and autumn could be due to the fact that plant sources biosynthesize higher amounts of bioactive compounds during these seasons, in comparison to the rainy seasons (summer and winter) in the Sonoran Desert [49], pointing to plants belonging to Asteraceae family, such as *Ambrosia deltoidea* and *Encelia farinosa*, which were previously reported as contributing plants to propolis production throughout the Sonoran Desert [37,38].

## 5. Conclusions

In conclusion, these results indicate that CP is a source of bioactive compounds that induce a remarkable antiproliferative effect, as well as antioxidant activity and pharmacological potential, which are is clearly influenced by quantitative variations in the polyphenolic profile of CP throughout the year, mostly in poplar-type constituents. According to our results, the most bioactive collection time for CP is during the autumn and spring seasons. Further studies are needed to characterize the molecular basis of the bioactivity of CP.

## Figures and Tables

**Table 1:** Antiproliferative activity of seasonal samples of CP in M12.C3.F6 cells.

Seasonal CP	IC_50_ (µg/mL or µM)
Autumn	5.9 ± 0.6 ^a,b^
Winter	12.1 ± 0.5 ^b^
Spring	9.2 ± 1.3 ^a^
Summer	9.5 ± 0.4 ^a^
CAPE	1.4 ± 0.1

Seasonal samples of CP were obtained during the 2014–2015 period. All IC_50_ values represent the mean of three independent determinations performed by triplicate ± standard deviation. CAPE (caffeic acid phenethyl ester) was included as positive antiproliferative control. IC_50_ data are expressed in µg/mL or µM, according to propolis extract or CAPE, respectively. IC_50_ data were analyzed by one-way ANOVA followed by Tukey’s multiple comparison test. The antiproliferative effect of all seasonal CP methanolic extracts was significant, in comparison to the DMSO control (*p <* 0.05). ^a^ Value of *p <* 0.05 of significance from the corresponding IC_50_ to the IC_50_ of CP autumn treatment. ^b^ Value of *p <* 0.001 of significance from the corresponding IC_50_ to IC_50_ of CP autumn treatment.
